# Network Effects of Risk Behavior Change Following Prophylactic Interventions

**DOI:** 10.1371/journal.pone.0064653

**Published:** 2013-08-01

**Authors:** Rajmohan Rajaraman, Zhifeng Sun, Ravi Sundaram, Anil Kumar S. Vullikanti

**Affiliations:** 1 College of Computer and Information Science, Northeastern University, Boston, Massachusetts, United States of America; 2 College of Computer and Information Science, Northeastern University, Boston, Massachusetts, United States of America; 3 College of Computer and Information Science, Northeastern University, Boston, Massachusetts, United States of America; 4 Department of Computer Science and Virginia Bioinformatics Institute, Virginia Tech, Blacksburg, Virginia, United States of America; Northwestern University, United States of America

## Abstract

We formulated a network-based model to understand how risk behavior change in conjunction with failure of prophylactic interventions can lead to unintended outcomes where “less (intervention) is more (effective).” Our model captures the distinction between one- and two-sided risk behavior change. In one-sided situations (e.g. influenza/H1N1) it is sufficient for either individual in an interaction to exhibit risk behavior change whereas in two-sided situations (e.g. AIDS/HIV) it is necessary for both individuals in the interaction to exhibit risk behavior change, for a potential transmission of the disease. A central discovery is that this phenomenon occurs at differing levels of intervention coverage depending upon the “sidedness” of the interaction. We find that for one-sided interactions, sufficiently high vaccination coverage is necessary for mitigating the effects of risk behavior; for two-sided interactions, it is essential to combine prophylactic treatments with programs aimed at reducing risky behavior. Furthermore, again dependent on the “sidedness,” targeting highly connected nodes can be strictly worse than uniformly random interventions at the same level of coverage.

## Introduction

For many diseases, such as influenza and HIV, interventions using anti-virals and vaccinations are commonly used to control the spread of the diseases, and are usually universally recommended, barring individual constraints. Recent studies have shown significant benefits of anti-retrovirals for reducing the spread of HIV [1]. Such vaccination or anti-viral treatments have very widely varying levels of efficacy (25–75% in the case of HIV [1],[2], 80–85% in the case of Pertussis [3], and between 10–80% in the case of influenza [4], depending on the demographics and the specifics of the flu strain). Most studies about the efficacy of such interventions do not take individual behavior into account in a realistic manner, though many researchers have pointed out that it is an important issue, e.g., [5]. Systematic research and data analysis on this aspect are limited. Petrie et al. [6] observe that people are not very well aware of this limitation, and studies often over-estimate the efficacy of vaccines. Further, Reiber et al. [7] have recently documented significant changes in behavior in vaccinated individuals (possibly because of perceived protection offered by the vaccination), leading to an increase in contact by a treated individual; such a behavioral change following vaccination could also be a natural evolutionary response [8], [9]. Regardless of the underlying reasons, failure of prophylactic interventions in conjunction with increased social behavior can have significant unexpected effects on the disease dynamics. In a series of important papers [10], [11] Blower and her collaborators demonstrated that risk behavior change, in the context of HIV vaccinations, could lead to unintended outcomes. Subsequently, several independent studies have confirmed this phenomenon of perversity in the use of HIV vaccines and anti-virals, and vaccines for the human papillomavirus (HPV) [2], [5], [12]–[21]. The phenomenon of an increase in risky behavior following protection is also referred to as “moral hazard” and has been studied extensively in a number of areas, such as insurance (e.g., [22]); in the epidemiology literature, this is referred to more commonly as “risk behavior” (e.g., [10]), and we will adopt this terminology for most of the paper.

A fundamental question in mathematical epidemiology is to determine the fraction of the population that needs to be vaccinated or treated with anti-virals in order to minimize the impact of the disease, especially when the supply is limited. Modern epidemiological analysis is largely based on an elegant class of models, called SIR (susceptible-infected-recovered), which was first formulated by Reed and Frost in the 1920 s, and developed over the subsequent decades. The SIR model and its variants have been highly influential in the study of epidemics [11], [23]–[27]. These models, however, do not attempt to capture the rich structure of the contact network over which interactions occur. Network structure has a direct effect on both the spread of diseases as well as the nature of interactions, which has been observed by a number of researchers, e.g. [28], [29]. In the emerging area of contact network epidemiology, an underlying contact graph captures the patterns of interactions which lead to the transmission of a disease [30]–[37]. Many studies have predicted the spread of diseases through networks using mathematical analysis or simulations. As we have argued above, moral-hazarding/risky behavior clearly plays an important role in the effectiveness of such interventions. While the impact of risky behavior on prophylactic treatments has been studied in previous work, the extent of the perversity and its dependence on network structure as well as the precise nature of the behavior change has remained largely unknown. Similar issues arise in the context of the spread of malware through infected computers. Several studies, e.g., [38], have found that computer and smart-phone users do not relate bot infections to risky behavior, such as downloading spam mails, though a large fraction of users have updated anti-virus software. It is plausible that such phenomena can also be associated with risky behavior in many cases.

In this paper, we study the impact of risk behavior change on the spread of diseases in networks and observe a rich and complex structure dependent both on the underlying network characteristics as well as the nature of the change in behavior. We use a discrete-time SIR model of disease transmission on a contact network, in which each edge has a certain probability of disease transmission. An infected node is assumed to recover in one time step. We consider both uniform random vaccination (where each node is vaccinated independently with the same probability) as well as targeted vaccinations (where nodes are vaccinated based on their degree of connectedness). Vaccines are assumed to fail uniformly and randomly. Though we focus on vaccinations and disease transmission, the basic results apply to other prophylactic treatments such as anti-virals, and other phenomena such as malware spread. We model risk behavior change by an increase in the disease transmission probability, *with no change in the contacts*. We emphasize here that the change in risk behavior is only a function of whether a node is vaccinated, and is independent of the failure of the vaccination. Any adverse impact of a risk behavior change, however, manifests only when the vaccination of the node that indulged in increased risky behavior fails.

A significant aspect of our work is the consideration of the “sidedness” of risk behavior change. We classify risk behavior as one-sided or two-sided based on whether the increase in disease transmission probability requires an increase in risk behavior of both the infector and the infectee or just the infector. As examples: influenza (H1N1) may be modeled as a one-sided disease since a vaccinated individual may be motivated to behave more riskily in terms of increased contact (e.g., going to crowded places, increasing travel on planes), thus increasing the chance of infecting all the individual comes in contact with; whereas AIDS (HIV) may be modeled as a two-sided disease since the increase in disease transmission requires both the individuals participating in the interaction to engage in risky behavior. Of course, these examples are simplistic and most diseases have elements of both one-sided as well as two-sided risk behavior. An important caveat is that in this study, we assume the network is static (i.e., the contacts remain unchanged, though their intensity is changed, which alters the transmission probability), and does not co-evolve with the behavioral change – we note that even this restricted setting is quite non-trivial and poorly understood, especially in the context of network effects.

Our main findings are threefold, and are described at a high level here. We discuss them in more detail in Section 0, after describing our model and simulation setup in Section 0. First, we find that the *severity of the epidemic often varies non-monotonically as a function of the vaccinated fraction*. The specific dynamics depend on the nature of risky behavior, as well as the efficacy of the vaccine and the contagiousness of the disease (the less reliable the vaccine, and more contagious the disease, the greater the non-monotonicity). Therefore, in general, we observe that increased vaccination rate does not immediately imply less severity; in some cases, the severity could increase by as much as a factor of two. Second, we find that *one-sided risk behavior change leads to unintended outcomes at low levels of vaccination, while two-sided risk behavior change leads to unintended outcomes at high levels of vaccination*. Our analysis indicates that effective prophylactic interventions against diseases with one-sided risk behavior change need to have sufficiently high coverage; on the other hand, for diseases with two-sided risk behavior change, it is essential to combine treatments with education programs aimed at reducing risky behavior. Our third and, perhaps, most surprising finding is that *interventions that target highly connected individuals can sometimes be worse than random interventions* for the same level of coverage and that this phenomenon occurs both for one-sided as well as two-sided risk behavior change. Given prior work on targeting vaccine distributions, this finding flies in the face of intuition that expects that targeted vaccination would confer greater benefits. Thus, our results offer new insights and directions for public policy on containing epidemic spread through prophylactic interventions.

## Comparison with Related Work

The study of spread of diseases and the ways to control them has been a major research topic for centuries. Modern epidemiological analysis is largely based on an elegant class of models, called SIR (susceptible-infected-recovered), which was first formulated by Reed and Frost in the 1920s, and developed over the subsequent decades. The SIR model and its variants have been highly influential in the study of epidemics [11], [23]–[27]. These models, however, do not attempt to capture the rich structure of the contact network over which interactions occur. Network structure has a direct effect on both the spread of diseases as well as the nature of interactions, which has been observed by a number of researchers, e.g. [28], [29]. In the emerging area of contact network epidemiology, an underlying contact graph captures the patterns of interactions which lead to the transmission of a disease [30]–[37]. Many studies have predicted the spread of diseases through networks using mathematical analysis or simulations.

Implications of risk behavior in public health have been examined earlier. In particular, the counterintuitive impact of vaccination owing to risky behavior was first discovered by Blower and McLean [10], [11], [39] in the case of HIV. Subsequently, several independent studies have confirmed this phenomenon and have offered potential explanations [12]–[15]. However, the extent of the perversity and its dependence on network structure as well as the precise nature of the behavior change has remained largely unknown. Our paper will go some way towards the resolution of these issues.

The analyses conducted in prior studies of risk behavior in public health are based on differential equation models, which divide the population into a fixed set of groups and model the interaction between different groups in a uniform way. The epidemic spread is then characterized by the “reproductive number,” denoted by 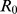 (see [40] for a discussion on 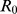 in static and dynamic networks), with the expected epidemic size exhibiting a threshold behavior in terms of 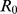. In contrast, we use a network model that captures the fine structure of interactions between (an arbitrary number of) individuals rather than (a fixed set of) groups, and find that the network structure has a significant impact on the resulting dynamics. The heterogeneous network model extends to a larger range of real-life situations but the increased fidelity comes at a price. The outcomes are more complicated and varied and the general approach of lowering 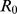 does not appear to be directly applicable. Another new contribution of our study is the focus on the sidedness inherent in risk behavior change, which has not been considered before. Prior research has implicitly assumed one-sided risk behavior change where vaccinated individuals engage in risky behavior increasing the chances of infection of those they come in contact with. This work explicitly treats both one-sided and two-sided risk behavior changes and shows that their differing impact needs to be considered in public intervention policies.

## Our Model

**Figure 1:**
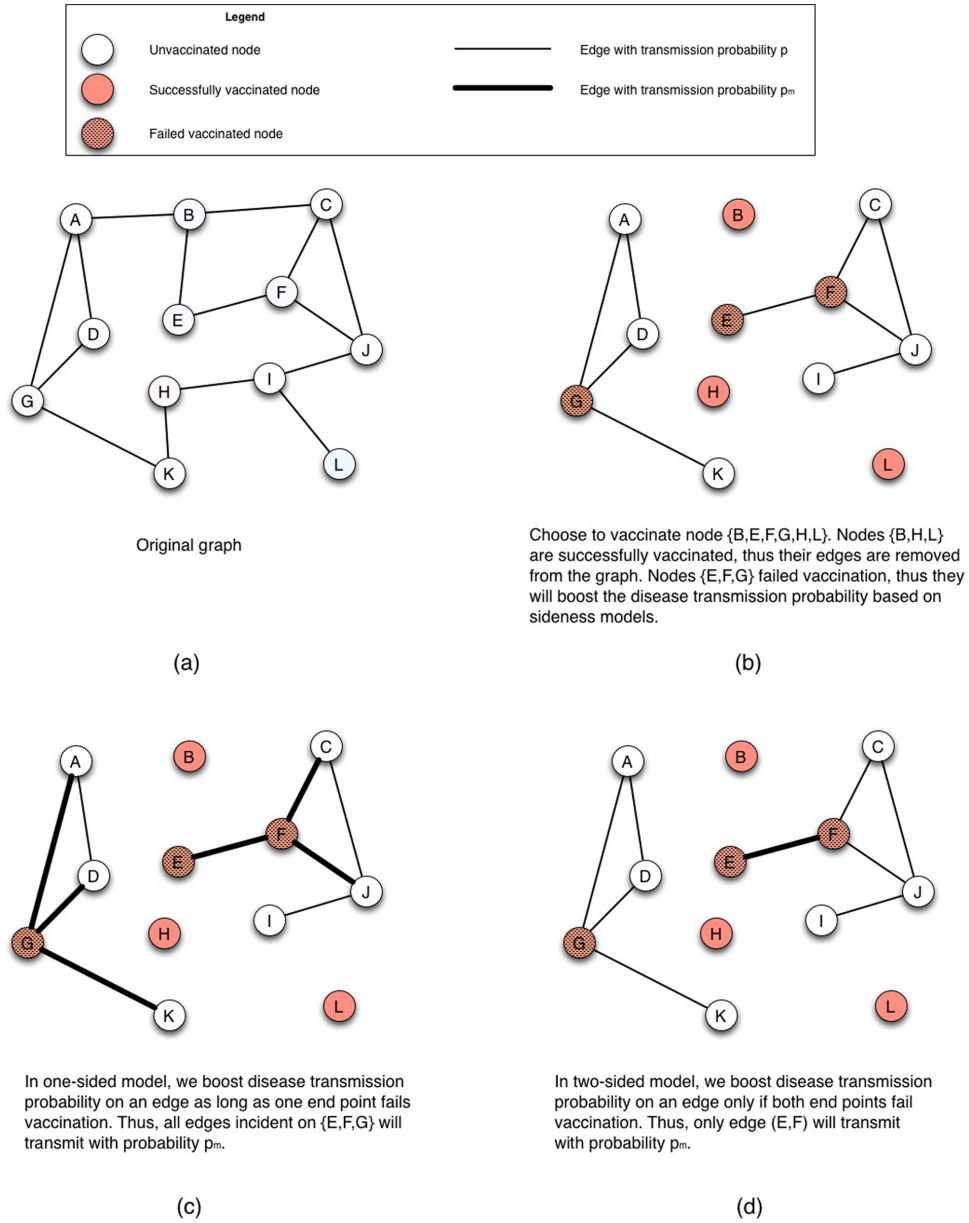
Sidedness of risk behavior change: the one-sided and two-sided models.

We obtain our results through both analytical techniques and simulations on a range of networks including preferential attachment networks [41] and large synthetic and real-world networks. For our theoretical analyses, we adopt the SIR model of epidemics defined on networks. Let 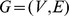 denote an undirected social contact graph, where 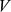 denotes a set of people (referred to as nodes henceforth) and 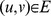 denotes a contact between nodes 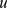 and 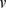 (see Figure 1(a) for an example). If node 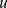 becomes infectious, it will infect each of its susceptible neighbors independently with probability 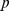 (referred as *base transmissivity*). Each node in the graph is either vaccinated (e.g., nodes 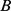 or 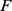 in Figure 1(b)) or not (e.g., nodes 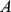 or 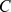 in Figure 1(b)). If a node 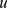 is not vaccinated, we label it as UV. The vaccine fails with probability 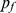; for convenience, we also define vaccine success probability 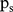 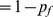. If a node 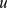
's vaccine fails, we label it as VF; otherwise, we label it VS. Both UV and VF nodes are susceptible. We assume that vaccine failure is a stochastic event and that (vaccinated) nodes do not know if (their own) vaccination succeeded or not. When a node is vaccinated, its risk behavior changes; i.e., it increases its contact to some of its neighbors. If the vaccination is successful, this increase in risky behavior has no impact on disease transmission. If the vaccination fails, however, then the increased contact results in *boosted transmissivity* 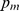 along the contact edges – in the one-sided model a node infects all its susceptible neighbors with boosted transmissivity 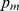, while in the two-sided model, it only infects those neighbors with boosted transmissivity 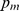 that have also had a failed vaccination. In the rest of the paper, we use 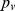 to denote the probability that a node is vaccinated, under a campaign of uniformly random vaccination. For easy reference, we list the different probability parameters in Table 1.

**Table 1:** Probability parameters.

Notation	Parameter
*P*	base transmissivity, the probability of disease transmission along an edge
*p_m_*	boosted transmissivity, the transmission probability following risk behavior change
*p_v_*	probability that a given node is vaccinated
*p_f_*	probability that the vaccination of an arbitrary node fails
*p_s_*	vaccine efficacy ( = 1−*p_f_*)

The disease transmission process is thus defined by the tuple 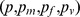 in the following manner: every node is labeled with UV,VS VF, with probability 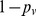, 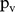 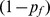, and 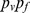, respectively. All nodes labeled are removed from the graph. Each edge 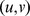 connecting two surviving nodes 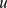 and 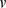, is “open” (or retained in the graph, in the language of percolation, which corresponds to disease transmission on this edge), or “closed” (or removed from the graph), with some probability depending on the model – (i) in the one-sided model, edge 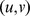 is open with probability 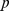, if both 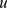 and 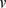 are labeled UV, and is open with probability 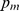 if one of 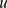 and 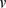 is labeled VF; (ii) in the two-sided model, edge 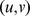 is open with probability 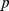, unless both 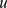 and 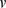 are labeled VF. Following the well known correspondence between bond percolation and disease transmission, the connected component containing a specific node 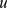 is the (random) subset of nodes infected, if the disease starts at 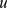. If the components resulting from one random instance of the above stochastic process are 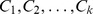, then 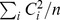 denotes the expected outbreak size of the disease starting from a random initial node. In our analysis, we use this as a measure of *epidemic severity*.

## Results and Discussion

We summarize our analytical and simulation based findings that both one-sided and two-sided behavior changes can lead to unintended outcomes (less vaccination is more effective) across a wide range of contact networks. These are discussed in greater detail in Sections Simulation Results for Diverse Networks and Analytical Results for the Erdös-Rényi Model, respectively.

### 1. Simulation results in a wide range of networks

We study the impact of risky behavior in a wide range of networks, including random graph models (such as the Erdös and Rényi [42] and Preferential attachment [41] models), and realistic networks, such as social contact networks and peer-to-peer networks. We find that one-sided behavior change leads to unintended outcomes at low levels of intervention, in which the epidemic severity increases with 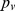, up to a point, while two-sided behavior change leads to unintended outcomes at high levels of intervention, in which the epidemic severity starts increasing beyond a threshold value of 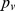. Further, we find that targeted vaccination can be strictly worse than random vaccination for some level of vaccine coverage, and this phenomenon occurs both for one-sided as well as two-sided behavior change. In the literature it has been observed that targeting highly connected individuals for vaccination lead to better outcomes as opposed to random coverage [31], [36], [43]. Our finding adds nuance to the existing results when risky behavior is taken into account. This counterintuitive phenomenon can also be explained by the tug of war between successful vaccination and risky behavior. If the effect of risky behavior is dominant then one would expect that targeted vaccination ends up being worse than random coverage since it is the targeted high-degree individuals that are the most responsible for creating additional contagion. And, in fact the evidence supports this explanation in that we see targeted coverage being inferior to random coverage at low levels of vaccination in the one-sided case but at high levels in the two-sided case.

### 2. Analytical results

We mathematically establish the phenomena of perversity and non-monotonicity for graphs generated according to the Erdös-Renyi model [28], denoted by 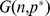, in which each edge between a pair of nodes is chosen independently with probability 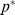. We prove rigorously that there exist 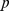, 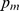, and 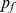, such that (i) in the one-sided model, it almost surely holds that the epidemic severity is 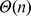 for both 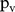 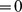 and 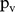 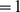, yet 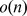 for some 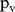 in 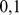; (ii) in the two-sided model, the epidemic severity is 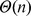 for both 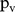 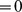 and 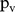 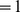, yet 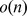 for some 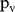 in 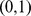. This implies that there is a choice of parameters (which turns out to be be quite broad), such that as the vaccinated fraction 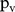 is varied, the epidemic severity shows a non-monotone behavior.

In [44], we show that the phenomenon of perversity exists in a broad class of graphs. Specifically, we consider *locally finite graphs*, which have been widely studied in percolation theory (e.g., see [45]). Locally finite graphs include infinite graphs in which each node has bounded degree. Using techniques from percolation theory, we show that in every locally finite graph 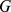, there exist 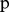, 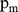, and 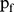, such that: (i) the epidemic severity is finite for both 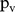 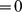 and 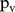 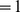, yet infinite for some 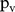 in 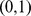 in the one-sided model; (ii) the epidemic severity is infinite for both 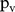 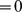 and 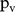 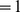, yet finite for some  in 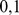 in the two-sided model. This result (which is discussed in [44], since it is beyond the scope of this paper) provides strong evidence of the universality of the phenomenon and begs for a natural and intuitive explanation. Our best structural understanding at this point is that this is the consequence of two competing tensions – vaccine success that serves to contain the spread and risky behavior that, exacerbated by vaccine failure, serves to boost the contagion. In the one-sided situation since it is sufficient for infection spread to have just the one party in an interaction exhibiting risky behavior we see perversity manifesting itself at low levels of vaccination. Whereas, in the two-sided situation since it is necessary for both the interacting parties to exhibit risky behavior we see perversity manifesting itself only at high vaccination levels which is a prerequisite for a non-trivial fraction of parties with failed vaccines to exist.

## Simulation Results for Diverse Networks

In this section, we examine the impact of risk behavior in a wide range of networks. Recall from Section that the disease transmission is a random process, defined by the parameter set (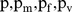). We study the epidemic severity for the networks described in Table 2, and our main goals are to study empirically: (i) the extent of unintended outcomes, and (ii) the impact of target vaccination strategies in the one-sided and two-sided scenarios. For each network in Table 2, we run simulations over wide range of parameter set (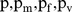). Our main results are summarized below.

**Table 2:** Descriptions of the networks used in the paper.

name		*n*	*m*	description
Human contact	NewRiverValley [50]	74,375	1,888,833	Synthetic human contact network for New River Valley county in Virginia.
Social communication	Enron mail [51], [52]	36,691	367,666	Email communication network in a company.
Peer-to-peer network	Gnutella [53], [54]	10,876	39,994	Gnutella peer-to-peer file sharing network from August 2002
Random graphs	Preferential attachment [41]	100,000	300,000	Generated using Python NetworkX library.
	Erdös and Rényi [42]	100,000	5,000,000	

For each network we show its type, name, number of nodes 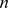 and edges 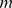.

- We find that the unintended outcome in both the one- and two-sided settings increase with the boosted transmissivity 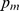. This seems to be uniformly true for all the networks we study.
- The unintended outcomes in both the settings increases with the vaccine failure rate in all the networks.
- Targeted vaccination strategies (based on degree) can be strictly worse than random vaccination for various parameter values. This is an important observation, since targeted interventions have been strongly advocated.

We discuss our simulation based results in more detail below. For simplicity, we show the results for each network mentioned in Table 2 separately, but summarize the main observations below. In order to capture real disease transmission through simulations, we find typical values of 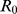, the basic reproduction number, for many diseases such as influenza and HIV [46]–[48]. Then, we divide 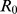 by the average degree of the network, and use it as the base transmissivity 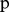. For vaccination success probability, we use the efficacy for real vaccines [1], [2], [4].

### 1. Impact of the boosted transmissivity 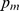


**Figure 2:**
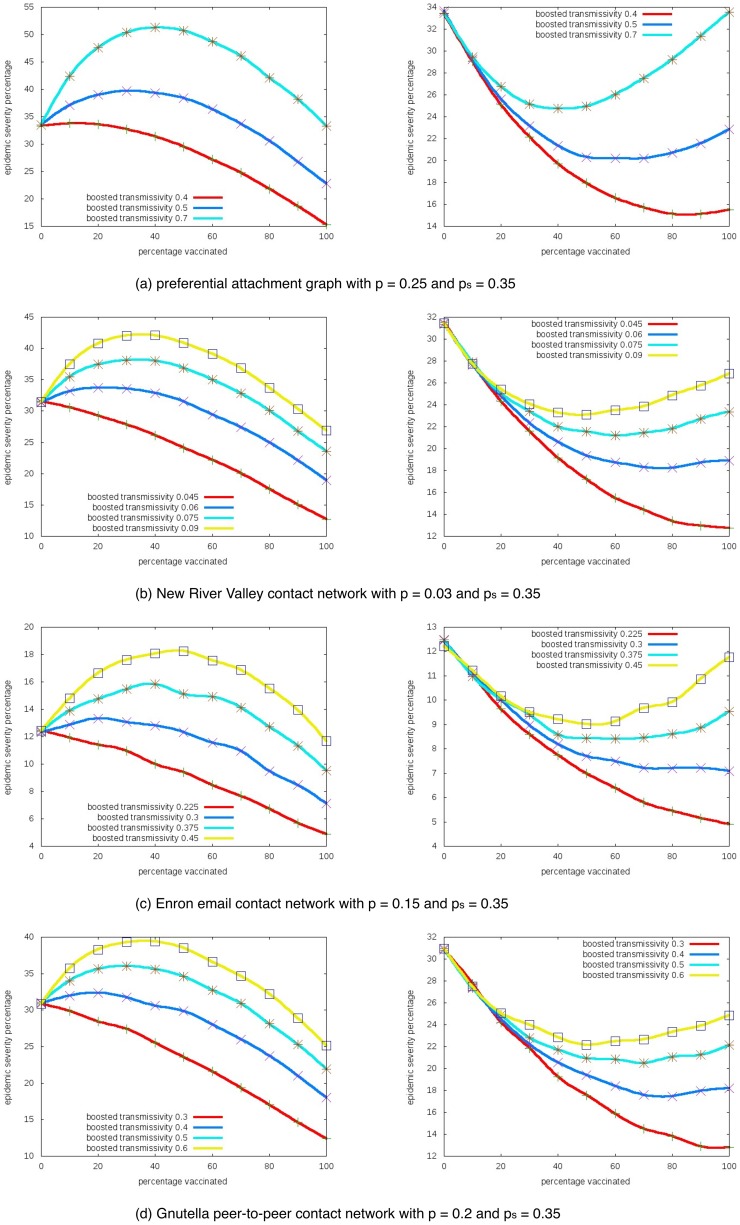
Impact of the boosted transmissivity parameter 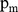. Epidemic severity with different boosted transmissivities in one-sided (left) and two-sided (right) risk behavior models. 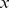-axis is the percentage of nodes taking interventions, and 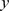-axis is the expected percentage of nodes getting infected.

Figure 2 shows how the change of boosted transmissivity affect the unintended outcomes for the different networks. The 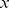-axis is 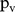 (percentage of vaccinated population) and the 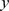-axis is the epidemic severity (expected percentage of nodes getting infected). We fix the base transmissivity 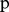 and the vaccination success probability 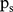, then plot the curves for different boosted transmissivity. As these results show, the unintended outcome increases with 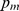 in both settings.

### 2. Impact of the base transmissivity

**Figure 3:**
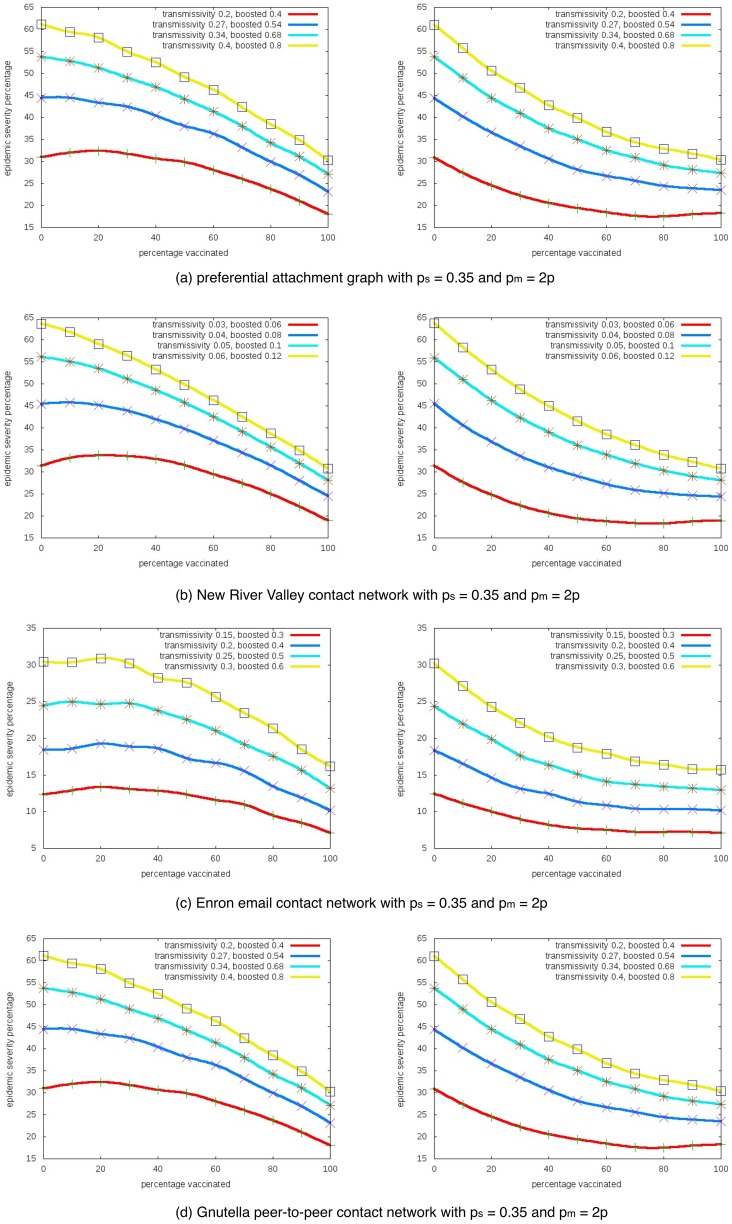
Impact of base transmissivity parameter 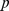. Epidemic severity with different transmissivities in one-sided (left) and two-sided (right) risk behavior models. 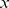-axis is the percentage of nodes taking interventions, and 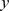-axis is the expected percentage of nodes getting infected.

Figure 3 shows how the change of base transmissivity affect the unintended outcomes. The 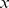-axis is 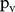 and the 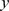-axis is the epidemic severity. We fix the vaccination success probability 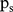 and keep the boosted transmissivity 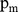 twice the base transmissivity 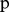 (i.e. 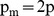), then plot the curves for different base transmissivity.

### 3. Impact of the vaccination efficacy

**Figure 4:**
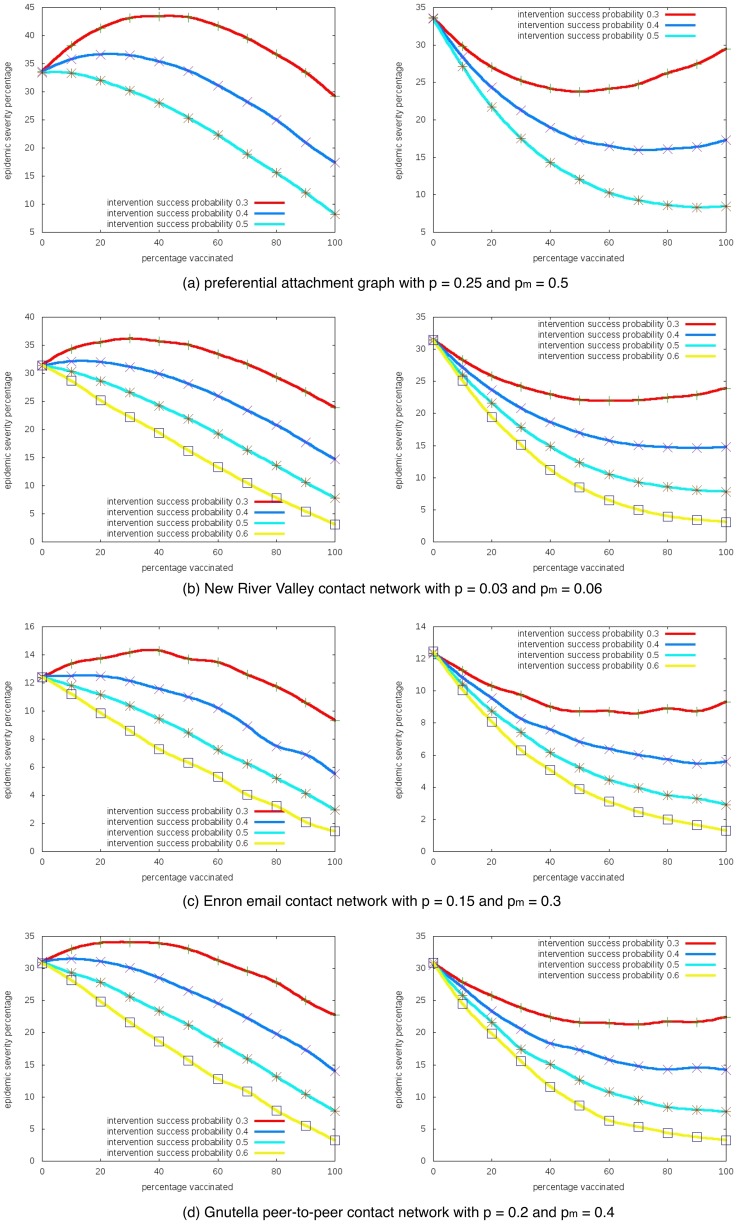
Impact of the vaccination efficacy parameter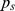. Epidemic severity with different intervention success probabilities in one-sided (left) and two-sided (right) risk behavior models. 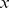-axis is the percentage of nodes taking interventions, and 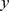-axis is the expected percentage of nodes getting infected.

Figure 4 shows how the change of vaccination success probability affect the unintended outcomes. The 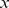-axis is 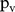 and the 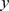-axis is the epidemic severity. We fix the base transmissivity 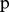 and the boosted transmissivity 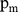, then plot the curves for different vaccination success probability. We observe that the perversity increases with the vaccination failure rate.

### 4. Targeted vs random interventions

**Figure 5:**
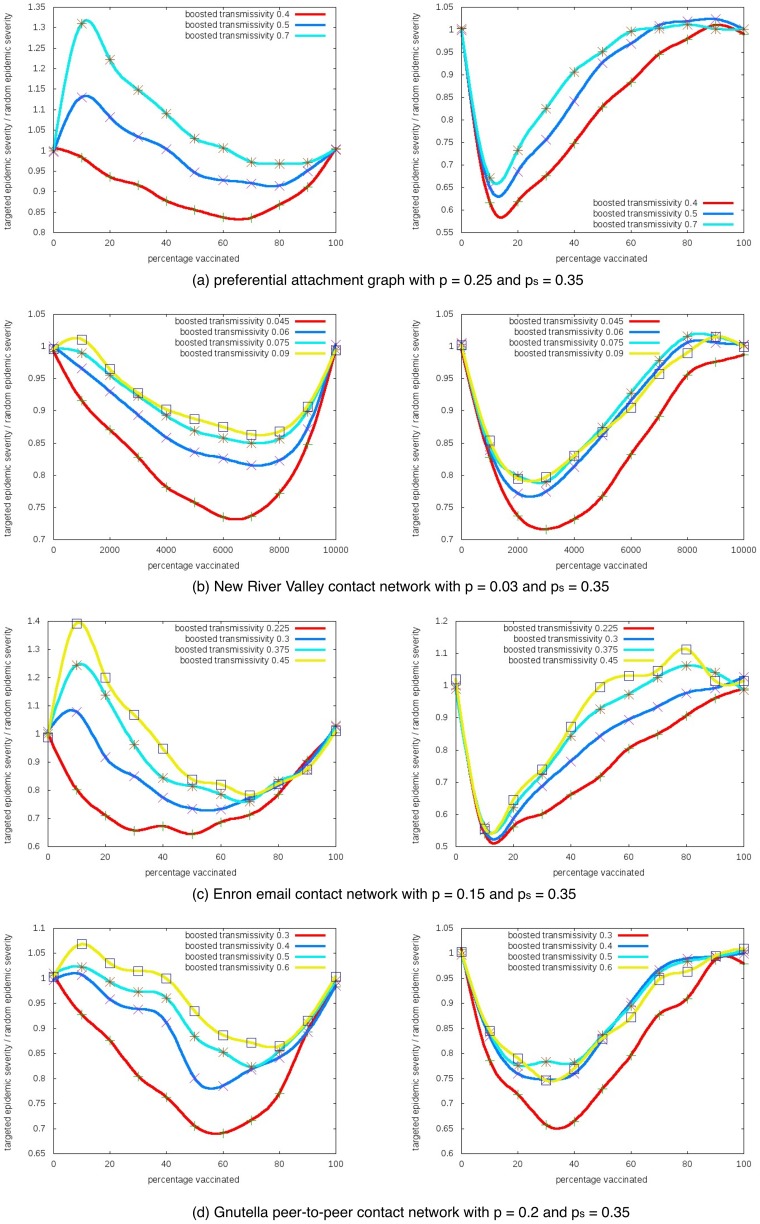
Targeted vs random interventions. Epidemic severity comparison of random and targeted intervention strategies in one-sided (left) and two-sided (right) risk behavior models. 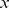-axis is the percentage of nodes taking interventions, and 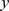-axis is the ratio of the epidemic severity in targeted intervention strategy and the epidemic severity in random intervention strategy.

Figure 5 shows that the efficacy of targeted vaccination (relative to random vaccinations) can vary significantly in the presence of such one-/two-sided behavior. For various parameter choices, targeted vaccination can even be worse. The 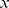-axis is 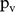 and the 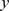-axis is the ratio between the epidemic severity under targeted vaccination strategy and the epidemic severity under random vaccination strategy. If 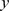 value is bigger than 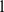, it means targeted strategy is worse than random strategy. We fix the base transmissivity 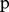 and the vaccination success probability 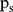, then plot the curves for different boosted transmissivity.

## Analytical Results for the Erdös-Rényi Model

In this section, we give formal proofs of perversity and non-monotonicity in Erdös-Rényi random graphs. We have observed the non-monotonicity is pervasive in wide range of contact graphs, including scale-free graphs, Erdös-Rényi graphs, and other synthetic or real world graphs. Theorem 3 gives the rigorous proof of one-sided model and two-sided model for Erdös-Rényi random graphs. We establish two key lemmas that apply to complete graphs, from which we derive the results for random graphs.

### Lemma 1

Given a complete graph as the contact network, for intervention with any success probability 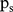, there exists parameter set 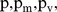 such that there is non-monotonicity in two-sided risk behavior model.

#### Proof

We set the disease transmission probability 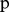 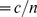 where 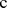 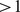. Let 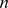 be the number of nodes in the network. We first show that the expected epidemic size is 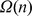 in two extreme cases: when nobody takes interventions, and when everybody takes interventions. We then show that there exists an intermediate vaccination point, where the expected epidemic size is 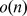, thus establishing non-monotonicity.

When nobody takes interventions, then the impact of disease transmission is captured by an Erdös-Rényi graph over 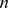 nodes in which each edge exists independently with probability 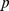. By [42], there is a giant connected component with high probability (size of the connected component is 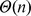), implying a 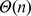 bound on the expected epidemic size.

When everybody takes interventions, 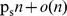 nodes will have successful interventions with high probability, and thus removed from the graph. The remaining 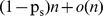 nodes will all exhibit risk behavior changes. Thus, the disease transmission probability between each pair of nodes is 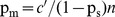, where 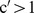. By [42], there is a giant connected component with high probability (size of the connected component is 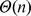), again implying a 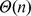 bound on the expected epidemic size.

Now we are going to show there exists a 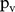, such that, if we apply interventions to each node independently with probability 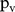, the epidemic severity will be 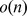 with high probability. Let 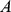 be the set of nodes that haven't taken interventions, 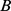 be the set of nodes that have taken interventions but failed, and 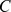 be the set of nodes that have taken interventions and succeeded. 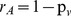 represents the probability of a random node being in set 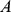, 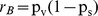 represents the probability of a random node being in set 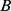, and 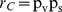 represents the probability of a random node being in set 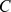. In the two-sided model, disease transmits with probability 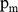 between nodes in set 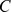, and 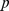 otherwise. Set 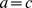 and 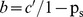. Let
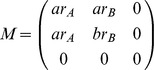



This yields a model of inhomogeneous random graphs with 3 types of vertices (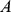, 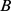, and 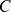). By [49] Theorem 1, if all the eigenvalues of 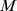 are less than 1 in absolute value, then the size of the largest connected component is 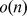. Let 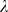 be the eigenvalues of 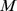.
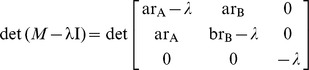


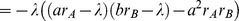


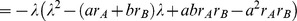



Solving 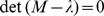, we have
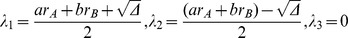
where 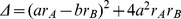. Since 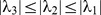, it is sufficient to show there exists a set of parameters that yields 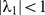. Set 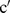 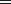 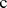.



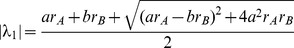


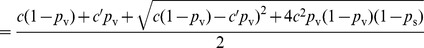


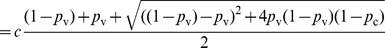


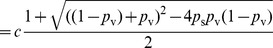


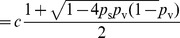
When 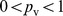, 
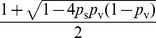
 is a constant smaller than 1. We can find 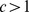 that satisfies 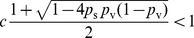. Thus, for intervention with success probability 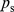, there exist parameters 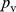, 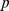, and 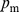, such that the epidemic size is 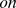. This completes our proof of this lemma.

### Lemma 2

*Given a complete graph as the contact network, for intervention with any success probability 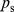, there exists parameter set 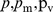, such that there is non-monotonicity in one-sided risk behavior model*.

#### Proof

We set the disease transmission probability 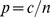 where 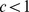. Let 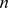 be the number of nodes in the network. We first show that the expected epidemic size is 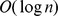 in two extreme cases: when nobody takes interventions, and when everybody takes interventions. We then show that there exists an intermediate vaccination point, where the expected epidemic size is 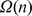, thus establishing non-monotonicity.

When nobody takes interventions, then the impact of disease transmission is captured by an Erdös-Rényi graph over 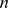 nodes in which each edge exists independently with probability 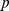. By [42], the size of the largest connected component is 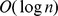 with high probability, implying an 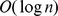 bound on the expected epidemic size.

When everybody takes interventions, 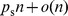 nodes will have successful interventions with high probability, and thus removed from the graph. The remaining 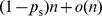 nodes will exhibit risk behavior changes. Thus, the disease transmission probability between each pair of nodes is 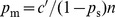, where 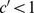. By [42], the size of the largest connected component is 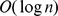 with high probability, again implying an 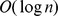 bound on the expected epidemic size.

Now we are going to show there exists a 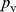, such that, if we apply interventions to each node independently with probability 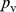, the epidemic severity will be 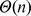 with high probability. Let 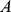 be the set of nodes that haven't taken interventions, 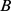 be the set of nodes that have taken interventions but failed, and 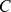 be the set of nodes that have taken interventions and succeeded. 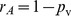 represents the probability of a random node being in set 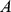, 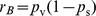 represents the probability of a random node being in set 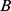, and 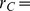 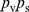 represents the probability of a random node being in set 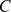. In the one-sided model, disease transmit with probability 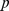 between nodes in set 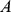, and  otherwise. Set 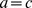 and 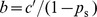. Let
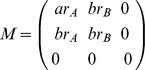



This yields a model of inhomogeneous random graphs with 3 types of vertices (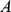, 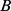, and 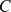). By [49] Theorem 1, if some eigenvalue of 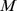 is larger than 1, then the size of the largest connected component is 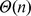. Let 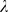 be the eigenvalues of 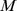.
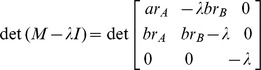


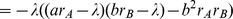


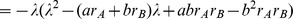



Solving 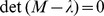, we have
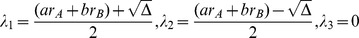
where 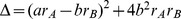. Since 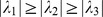, it is sufficient to show there exists a set of parameters that yields 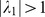. Let 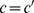.



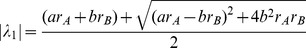


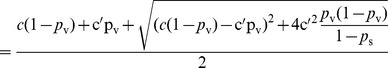


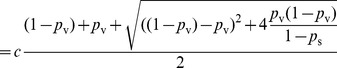


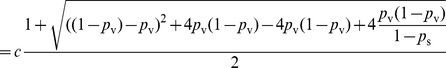


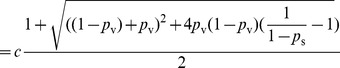


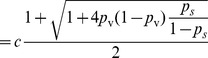
When 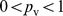, 
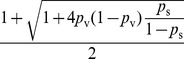
 is a constant greater than 1. We can find 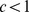 that satisfies 
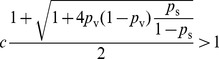
. Thus, for vaccination with success probability 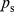, there exist parameters 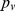, 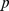, and 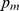, such that the epidemic size is 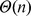. This completes our proof of this lemma.

### Theorem 3

For the Erdös-Rényi random graph model 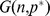, there exist 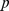, 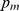, and 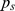, such that (i) in the one-sided model, it almost surely holds that the epidemic severity is 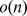 for both 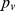 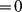 and 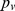 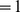, yet 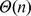 for some 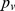 in 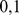; (ii) in the two-sided model, the epidemic severity is 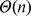 for both 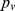 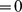 and 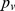 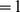, yet 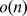 for some 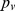 in 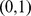.

#### Proof

We claim the disease transmission process on Erdös-Rényi random graph 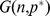 with parameter set 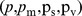 is the same as the disease transmission process on a complete graph with parameter set 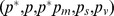. It's simply because the edge between each pair of nodes “opens” with the same probability in both random processes. Thus, for any disease transmission process on Erdös-Rényi random graph, we can reduce it to the corresponding process on a complete graph. Then by Lemma 0 and 0 we can conclude the statement of this theorem holds.

## Conclusion

In conclusion, risk behavior change in conjunction with failure of prophylactic interventions can have unintended non-monotone effects on the spread of diseases. This study has explicitly identified sidedness as an attribute of risk behavior change that needs to be taken into account in public policies for vaccinations and anti-viral treatments. For one-sided risk behavior change, it is imperative to have sufficiently high levels of coverage, while two-sided situations require both high coverage as well as programs aimed at reducing risky behavior. Our results echo the central premise of Blower-McLean that the development of efficacious prophylactic treatments and increasing their coverage need to go hand in hand with behavioral intervention strategies. These issues need to be revisited in the context of new anti-retroviral treatments being considered for HIV [1].
